# Genetic Profiling for Risk Reduction in Human Cardiovascular Disease

**DOI:** 10.3390/genes5010214

**Published:** 2014-03-12

**Authors:** Megan J. Puckelwartz, Elizabeth M. McNally

**Affiliations:** 1 Department of Medicine, University of Chicago, Chicago, IL 60637, USA; E-Mail: mjroy@uchicago.edu; 2 Department of Human Genetics, University of Chicago, Chicago, IL 60637, USA

**Keywords:** next generation sequencing, cardiovascular disease, genetic profiling, whole genome sequencing

## Abstract

Cardiovascular disease is a major health concern affecting over 80,000,000 people in the U.S. alone. Heart failure, cardiomyopathy, heart rhythm disorders, atherosclerosis and aneurysm formation have significant heritable contribution. Supported by familial aggregation and twin studies, these cardiovascular diseases are influenced by genetic variation. Family-based linkage studies and population-based genome-wide association studies (GWAS) have each identified genes and variants important for the pathogenesis of cardiovascular disease. The advent of next generation sequencing has ushered in a new era in the genetic diagnosis of cardiovascular disease, and this is especially evident when considering cardiomyopathy, a leading cause of heart failure. Cardiomyopathy is a genetically heterogeneous disorder characterized by morphologically abnormal heart with abnormal function. Genetic testing for cardiomyopathy employs gene panels, and these panels assess more than 50 genes simultaneously. Despite the large size of these panels, the sensitivity for detecting the primary genetic defect is still only approximately 50%. Recently, there has been a shift towards applying broader exome and/or genome sequencing to interrogate more of the genome to provide a genetic diagnosis for cardiomyopathy. Genetic mutations in cardiomyopathy offer the capacity to predict clinical outcome, including arrhythmia risk, and genetic diagnosis often provides an early window in which to institute therapy. This discussion is an overview as to how genomic data is shaping the current understanding and treatment of cardiovascular disease.

## 1. Introduction

Next generation sequencing has revolutionized the study of human genome variation and has the capacity to greatly influence health care decision making. The Human Genome Project, as conceived, was to sequence the first human genome in ~15 years at a cost of almost $3 billion using traditional dideoxy chain termination sequencing. In under 10 years, massively parallel next generation sequencing has led to the routine sequencing of exomes and whole genomes. Now achieved in weeks and at a cost that is multiple orders of magnitude less than the first genome, personalized genetic information is now widely available. These rapid advances in sequencing technology require new ways of collecting, analyzing, and disseminating genomic data. Herein, we discuss the ways that genomic information is currently being applied and how that data is shaping the ability to understand and treat cardiovascular disease (CVD).

Genetic variation is considered a contributory component for nearly all disease, whether single gene familial disorders or more common, complex traits with multiple gene involvement. Single gene or “Mendelian” disorders can be attributed to one gene as both necessary and sufficient to cause a large component of the disease phenotype. With complex traits, the gene-gene and gene-environment interactions are multifactorial. CVD consists of both single gene-familial disorders and common, complex disease. CVD is a major health concern affecting over 80,000,000 people in the U.S. alone [1]. CVD extends to heart failure and cardiomyopathy, heart rhythm disorders, atherosclerosis and thromboembolic events, aneurysm and others disorders. Familial aggregation and twin studies demonstrate that most, if not all, of CVD is heavily influenced by a genetic component [2,3,4].

## 2. Genetic Variation in CVD

Beginning in the 1980s, family-based linkage analysis was used to identify regions of the genome responsible for monogenic disease. The success of these methods required large families with penetrant phenotypes. Polymorphic genetic markers segregated with the phenotype of interest in large multi-generational families to identify chromosomal regions bearing the causal genes of interest [5]. Such familial linkage studies were highly successful in identifying genes for multiple forms of CVD. In 1989, linkage analysis defined the chromosomal location responsible for hypertrophic cardiomyopathy [6]. The next year, this data was used to identify mutations in the causative gene, *MYH7*, encoding β-myosin heavy chain [7]. Genetic determinants for Long QT syndrome, multiple cardiomyopathies, Marfan’s disease, and forms of congenital heart disease were identified highlighting both extensive locus and allelic heterogeneity [8,9,10,11,12,13,14]. However, these methods remain limited by the need for large families, a feature often not available since CVD confers survival disadvantage. Furthermore, much of CVD is under the influence of multiple genetic loci, and therefore requires alternative statistical methods and larger phenotypically and genetically characterized cohorts [15]. The HapMap project annotated the location of millions of single-nucleotide polymorphisms (SNPs) and took advantage of the long haplotype structure of the human genome [16]. Concurrently, commercially available platforms such as SNP arrays were developed that allowed simultaneous sampling of hundreds of thousands of SNPs paving the path for genome-wide association studies (GWAS). GWAS, which correlates SNPs with disease phenotypes, does not require a specific mode of inheritance and takes advantage of the extensive linkage disequilibrium (LD) in the human genome. In order to have enough statistical power to detect correlation, these large-scale association studies typically assess thousands to millions of SNPs across the genome in hundreds to thousands of cases and controls. According to the National Human Genome Research Institute (NHGRI) Catalog of Published Genome-Wide Association Studies [17] over 2800 strong SNP associations have been identified (*p* < 1 × 10^−8^) to date, and many of these are CVD-associated traits.

### 2.1. The Overlap between GWAS Hits and Monogenic Disease in CVD

CVD related phenotypes are well suited for GWAS because many CVDs have readily quantifiable discernable traits. Intriguingly, many GWAS “hits” overlap considerably with the same genes already linked to the disease though familial linkage studies. For example, Newton-Cheh and colleagues conducted a meta-analysis of three GWAS from ~14,000 individuals to examine the duration of QT interval from surface electrocardiograms [18]. QT duration reflects electrical depolarization and repolarization of the cardiac ventricles. A long QT interval is a biomarker for arrhythmias and a risk factor for sudden death. Non-familial QT disorders are still highly heritable (h^2^ ≈ 0.35), indicating a genetic component. GWAS identified 10 loci with significant (*p* < 5 × 10^−8^) association with QT interval. Five loci were those known to be involved in Mendelian long-QT syndromes, while the other five loci were genes that offer additional insights into variation at the QT interval. In total, the variation at these 10 loci accounted for 5.4%–6.5% of variation in the QT interval, which is quite high by GWAS standards. Genetic testing for the Mendelian form of long QT currently identifies mutations in ~75% of probands, so the additional GWAS loci may represent new candidate genes for mutation screening in familial long QT disorders.

*BAG3* (B-cell lymphoma 2-associated athanogene 3) is another example of GWAS results informing rare, Mendelian disease. In 2011, Villard and colleagues performed GWAS to identify loci contributing to sporadic dilated cardiomyopathy. Dilated cardiomyopathy (DCM) is exemplified by left ventricular dilation and systolic dysfunction, and is a major cause of heart failure and the principle indicator for heart transplant [19]. DCM has a high heritable component with 20%–35% of DCM patients having an affected first-degree relative [20]. More than 50 genes have been implicated in familial monogenetic DCM [21,22,23]. GWAS was performed with DNA from 1179 sporadic (non-familial) DCM patients and 1108 controls using ~500,000 SNPs [19]. The authors identified a DCM-associated non-synonymous SNP (p. C151R) in the coding region of *BAG3*. BAG3 is a co-chaperone that regulates HSP70 [24]. Further analysis of *BAG3* non-synonymous SNPs found another higher frequency SNP also associated with DCM. The authors investigated *BAG3* variation in familial DCM based on both the apparent association of *BAG3* non-synonymous SNPs with sporadic DCM and previously reported linkage with familial DCM between markers on chromosome 10 that include the *BAG3* locus [25]. In a cohort of 168 cases from DCM families, the authors identified additional likely pathogenic mutations in *BAG3*. Features of DCM were identified in 16 of 18 mutation carriers in the cohort [19]. In the same year, Norton and colleagues identified *BAG3* mutations in familial DCM [26]. The authors also created a *BAG3* knockdown zebrafish model that recapitulated the DCM and heart failure found in patients [26]. Together, the GWAS and familial data implicate *BAG3* in DCM and indicate that genes can harbor common variation that influences risk of disease in sporadic cases and rare variation that accounts for familial disease.

### 2.2. The Missing Heritability of GWAS

Despite the overlap of GWAS findings with monogenic disease, GWAS associations often account for only a small proportion of genetic variation. Also, the vast majority of variants identified by GWAS does not explain the high heritability or reveal the causal mechanism for the cardiovascular phenotype in question [27,28,29]. Using GWAS, McPherson and colleagues identified an interval on chromosome 9q21 that consistently associated with coronary heart disease in more than 23,000 participants from 6 independent cohorts [30]. Homozygotes for the risk allele have a 30%–40% increased risk for coronary artery disease (CAD). This finding remains perplexing as this region on 9q21 has no annotated genes and is not associated with known CAD risk factors [30]. This same chromosome 9q21 region has also been associated with myocardial infarction in a GWAS with 4587 cases and 12,676 controls [31]. Homozygotes for the allele have 1.64 times increased risk for myocardial infarction compared to noncarriers. Despite the evidence that this region is important for CVD pathophysiology, no disease mechanism has yet been identified.

GWAS is ultimately based on the idea that common diseases are caused by common genetic variants, each with small effect. Through additive and interactive effects, in conjunction with the environment, GWAS variants explain disease [27,32,33]. Recently, the common disease common variant hypothesis has been called into question due to the observation of missing heritability [34,35]. Missing heritability refers to the proportion of genetic variance that is not explained by the effect of common variants identified by GWAS. Several explanations have been suggested to explain missing heritability. It is possible that the number of variants responsible for a trait has been significantly underestimated, and that many more yet identified variants with very small effect sizes must be discovered. Another possibility is the presence of rare variants with larger effect size. Such rare variants are undetectable using present day SNP arrays, which are biased towards SNPs with allele frequencies close to 50%. There is also a possibility that missing heritability arises from variation caused by structural variants in the genome, also difficult to detect with SNP arrays. Lastly, there is also the possibility that gene-gene interactions and gene-environment interactions are of major importance, but are not appropriately modeled by current methods. 

GWAS typically interrogate SNPs with a minor allele frequency (MAF) > 5% while largely ignoring variants with lower population-based frequencies (MAF: 0.5%–5%) and those SNPs that are rare (MAF < 0.5%). The rationale for ignoring these variants relates to the strong linkage disequilibrium (LD) in the human genome. The human genome has an estimated mutation rate of approximately 1.4 × 10^−8^ or approximately 40 new mutations per generation. Projected over the current population of ~7 billion, the world currently has 300–400 million new mutations this generation [36]. Within the coding region alone, there are ~13,000 nonsynonymous variants per genome [37,38]. The National Heart Lung and Blood Institute sponsored exome sequencing project (NHLBI ESP) sequenced ~15,000 human-protein coding genes in >2000 individuals [39]. This study revealed an abundance of rare variants that were often population specific, potentially offering some support that rare variation explains some component of missing heritability. Alleles that confer high risk of disease are subject to negative selection pressure and would not reach high population frequencies. However, many different rare alleles in the same genes or gene pathways would induce the same phenotype across the population despite the “rare” genetic etiology. Johansen and colleagues recently performed GWAS and resequencing to determine mutational burden of rare variants in individuals with hypertriglyceridemia *versus* control subjects [40]. Hypertriglyceridemia is polygenic in nature and confers risk for cardiovascular disease. In general, GWAS variants explain <10% of variation for lipid traits [41,42]. Loci associated with GWAS signals were assessed for rare variation by focusing on protein-coding sequences of four GWAS-associated genes [40]. The authors identified a significant number of rare variants in individuals with hypertriglyceridemia compared to controls. An additional more restricted study, which analyzed only rare variants unique to cases and removing all reported variants without functional deficits, also confirmed a greater mutational burden in cases compared to controls [40]. These data indicate that GWAS-identified genes may carry rare variation that contributes to the heritability of a complex trait, and rare SNPs with relatively large effects on common disease may not be identified by GWAS studies. The underlying assumption that genotypes can be inferred between common alleles in strong LD may be flawed. Inferring genotypes based on strong LD may misestimate variation across the genome. While haplotype structure may be maintained with several to many common SNPs still inherited together, rare variation may occur between these SNPs, reducing the predictive value of the common SNPs. This rare variation may be the result of a higher than expected mutation rate and/or population structure. 

### 2.3. Rare Variation as a Cause of CVD

Next generation sequencing (NGS) provides a method to identify rare genetic variation. Massively parallel, array-based sequencing dramatically reduced cost and increased efficiency of DNA sequencing. Depending on the platform, sequencing reads range from ~35 base pairs to up to ~1000 base pairs. As the generation of sequence has become far more facile with NGS, large-scale alignment and interpretation has become the rate limiting step. Bioinformatics tools are available for alignment to the referent genome and calling variants (reviewed in [43,44]). A number of efforts are currently underway to catalog human genetic variation. The 1000 Genomes project has sequenced 1092 individuals from 14 populations using a combination of low coverage whole genome sequencing and exome sequencing [45]. It is estimated that this dataset captures 98% of SNPs at a frequency of 1%, finding 38 million SNPs, 59% of which were novel [45]. The NHLBI ESP provided whole exome sequencing on >6500 individuals, including 180,000 exons in 23,000 genes. The ESP is derived from diverse, well-phenotyped populations, including the Atherosclerosis Risk in Communities (ARIC) study, the Coronary Artery Risk Development in Young Adults (CARDIA) study, the Cardiovascular Health Study, the Framingham Heart Study, the Jackson Heart Study, and the Multi-Ethnic Study of Atherosclerosis. The data from this project will examine the genetic contribution to early-onset myocardial infarction, low-density lipoprotein cholesterol, body mass index/type 2 Diabetes mellitus, blood pressure, and ischemic stroke.

These databases are not only providing a rich dataset to identify rare variation, but they are also providing better allele frequencies across diverse populations for alleles once thought to be rare and pathogenic. Jabbari and colleagues examined the NHLBI ESP database (*n* = 6503) for variants previously associated with Catecholaminergic Polymorphic Ventricular Tachycardia (CPVT) and compared the frequency of those variants with the expected prevalence of CPVT in the population [46]. CPVT is a rare, lethal, hereditary cardiac disease characterized by fatal ventricular arrhythmias in the absence of structural defects of the heart or abnormal electrocardiographic findings [47]. Eleven percent of variants previously associated with CPVT were found in the ESP population, corresponding to a 1:150 prevalence of CPVT in the population, much higher than the known 1:10,000 prevalence. The 1000 Genomes (1KG) database was evaluated for the presence of predicted and previously reported pathogenic variation in three genes associated with cardiomyopathy (*MYH7*, *MYBPC3* and *TTN*) [48]. Nine percent of the population was identified as having a pathogenic variant (9%), which exceeds population prevalence estimates for dilated and hypertrophic cardiomyopathy (0.04%–0.2%, respectively). Similar studies have been performed for other cardiovascular diseases including Brugada Syndrome, cardiac channelopathies and arrhythmogenic right ventricular cardiomyopathy [49,50,51]. Since in every case the predicted pathogenic variation vastly exceeds the known disease prevalence, it suggests that on their own, these variants are not sufficient to cause disease. Whether these variants represent “at risk” genotypes for developing milder forms of disease is not known and requires prospective studies.

## 3. The Genetics of Dilated Cardiomyopathy

Cardiomyopathy is marked by a morphologically abnormal ventricle and frequently is associated with heart failure. Cardiomyopathy is divided into four groups: dilated (DCM), hypertrophic (HCM), restrictive (RCM), and arrhythmogenic right ventricular (ARVC), and the most common mode of inheritance is autosomal dominant. Depending on screening methods, nonischemic DCM, defined as DCM not arising from myocardial infarct or ischemia, is familial in 25%–50% of cases [52]. Greater than 50 genes have been implicated in familial DCM, and the majority of mutations are phenocopies as there are no outward clinical signs that predict the specific gene mutation. Identification of the genetic cause of cardiomyopathy is clinically important because of the high incidence of sudden death and clinical progression, which can be medically managed. In 2007, genetic testing relied on commercially available panels that interrogated only 5 genes using traditional sequencing. In 2011, Meder and colleagues developed an array-based panel that enriched for coding regions of 29 known and 18 novel, potential cardiomyopathy genes, followed by next-generation sequencing [53]. Current panels now include >50 genes [54], and the estimated sensitivity in DCM for detecting a pathogenic mutation is under 50% (Partners Healthcare [55]).

### 3.1. Next Generation Sequencing Identifies TTN as a Major Contributor to DCM

Next-generation sequencing facilitated the screening of *TTN* for DCM-causing variants. The *TTN* gene includes >350 exons and encodes the giant sarcomere protein titin, which ranges in size from ~27,000 to ~33,000 amino acids depending on isoform, making it the largest human protein [56]. Together, two titin molecules span the sarcomere, providing both passive and active contractile forces [57,58,59,60]. Previous work has linked *TTN* to dilated cardiomyopathy in families, but extensive screening was limited due to its large size [61,62,63]. Herman and colleagues developed an array to capture *TTN* exons and sequence *TTN* in patients with DCM (*n* = 312) [64]. *TTN* truncating mutations accounted for approximately 25% of familial DCM, but had a minimal contribution to hypertrophic cardiomyopathy (~1%). Approximately 30% of *TTN* truncating variants identified were putative splice site disrupting mutations whose effect on function can be difficult to assess *in silico*.

### 3.2. Beyond Panel Based Sequencing for Cardiomyopathy and Beyond

Despite the inclusion of *TTN*, the sensitivity for detecting a DCM mutation remains at just under 50%. There are several explanations for the missing variation. First, there are likely novel genes not yet associated with cardiomyopathy; second, certain genetic variation may not be readily detectable with NGS and SNP analysis. For example, nucleotide repeat expansions and structural variation is not commonly determined by NGS, as analytic methods are biased toward SNP analysis and small insertions and deletions; Third, pathogenic variation may arise from combinations of pathogenic variation, and analysis is generally biased towards finding a single pathogenic variant. This bias reflects that most families with inherited cardiomyopathy have autosomal dominant inheritance; Fourth, pathogenic variation may be non-coding and at this point, these regions are not captured by gene panels. To combat these problems and provide a more comprehensive variant profile, whole exome sequencing (WES) and whole genome sequencing (WGS) are being applied to identify disease-causing variation for many different diseases (Table 1). WES interrogates the coding portion of the genome; approximately 1%–2% of nuclear DNA, although at higher coverage than-comparably priced WGS [65]. Interrogating only a small portion of the genome, as in WES, is less expensive than WGS, and it is currently the most-readily interpreted, as approximately 85% of Mendelian-disease causing mutations cause changes in the coding sequence of the genome [66]. WES relies on commercially available sequence-capture arrays to enrich for the coding subset of genomic DNA, followed by massively parallel, next-generation sequencing of the enriched fragments. The choice of exome kit is an important consideration as the exome is approximately 30 megabases, and exome capture kits interrogate anywhere from 50 to 100 megabases, depending on the provider. Most of the additional sequences are untranslated regions (UTRs) that may be important for disease pathogenesis. 

**Table 1:** Comparison of Panel, whole exome sequencing (WES) and whole genome sequencing (WGS).

	Panel	WES	WGS
Variation in Known Genes	yes	yes	yes
Novel Gene Identification	no	yes	yes
Structural Variation	no	limited	yes
Non-coding Variation	no	limited	yes
Repeat testing required if first pass negative	yes	yes	no

### 3.3. Exome Sequencing of Multiple Family Members Improves Identification of Pathogenic Variation

Campbell and colleagues used exome sequencing on three members of a large multi-generational family with classical DCM. After sequencing, variants were filtered by frequency and protein prediction algorithms [67]. Eight potentially causative mutations were shared across the three family members, significantly reducing the potential variants to be considered. Variants were tested for segregation across the other family members and only one variant segregated with disease, *TNNT2* R173W, a known cardiomyopathy gene. These data are particularly convincing as the variant segregates across all affected members of the family, including fourth-degree relatives [67]. The underlying assumption with this analysis is that a single variant accounts for disease in all family members, an assumption that may or may not hold true. In 2013, Wells and others used WES in a large, multi-generational family with DCM of unknown etiology [68]. The proband had undergone extensive unrevealing panel testing for DCM. The authors selected 3 distantly related affected family members for WES. Distantly related family members that are obligate carriers of the same mutation should share fewer variants by descent than closely related members, allowing for easier filtering of potentially causative variants. Variants were then filtered for rarity, functional significance, conservation and autosomal-dominant inheritance [68]. Variants were also prioritized using the VAAST tool, a probabilistic search tool that combines conservation, amino acid substitution chemistry, and frequency data to build a unified likelihood-framework to identify damaged genes and disease-causing variants [69]. Heterozygous, nonsense, nonsynonymous and splice site variants shared between the 3 affected candidates were filtered based on rare frequency in 1 KG and ESP, leaving 26 candidates for analysis. Wells and colleagues then compared these variants to variants identified in ~70 exomes previously sequenced by their laboratory. Variants identified in multiple exomes were removed, leaving 2 putative variants. This comparison allowed for removal of false positives that may be inherent to some sequencing platforms and variant calling pipelines. An *RBM20* variant was identified in an unrelated patient with familial-DCM, consistent with its role in causing disease. The authors confirmed segregation within the larger pedigree of the variant in *RBM20*, a recently identified cardiomyopathy gene [70], providing statistical support for *RBM20* causing DCM. It is interesting to note that this finding is largely based on frequency in both the general population and in a cohort already sequenced by the laboratory. The authors did perform filtering that considered evolutionary conservation (Genomic Evolutionary Rate Profiling) and functional effects (Polyphen2) but these tools did not reduce the list as extensively as population and cohort frequency combined (8 variants *versus* 2 variants, respectively [69,71,72]. These data indicate that exome sequencing followed by extensive filtering, in conjunction with segregation analysis can identify rare, DCM-causing variation in known cardiomyopathy genes.

### 3.4. Identifying Cardiomyopathy Modifier Loci Using Broad Based Sequencing

With large gene panels or WES/WGS additional, disease-modifying variation can be identified. Intra-familial variability including age of onset, severity and penetrance is a hallmark of DCM, but the loci that modify DCM phenotypes have not been well elucidated [73,74]. Roncarati and colleagues used WES to investigate clinical variability in an extended family with 14 subjects that included four family members with severe DCM that required heart transplant in early adulthood [75]. WES was performed on three severely affected and one unaffected family member. Variants were filtered for rarity, predicted pathogenicity and inheritance. The filtering process left a list of only 28 variants that where further filtered through the Human Phenotype Ontology project, which uses formal ontology to capture phenotypic information to identify relationships between different genes and phenotypes [76]. Through this analysis, the authors identified eight genes with variation associated with Mendelian disease [75], and two of eight *LMNA* and *TTN*, were known to cause DCM. A missense *LMNA* mutation, previously identified in an unrelated DCM patient, was confirmed in all affected family members [77]. A *TTN* variant was identified in five family members, four of whom were severely affected, while the fifth is likely too young to yet be symptomatic. Doubly heterozygous family members had a more severe clinical course than the *LMNA*-only family members, indicating that the *TTN* variant modifies the clinical progression [75]. This mutational stratification is expected to prove useful for assessing clinical risk and guiding treatment. Broad based sequencing through gene panels or WES/WGS is now positioned to outline the contribution of multiple variants to DCM development. 

### 3.5. WES/WGS Can Identify New Genes for Cardiomyopathy

In 2011, Theis and colleagues used genome-wide mapping and exome sequencing in a consanguineous family with autosomal recessive DCM [78]. Genome-wide linkage analysis was performed in nineteen family members and a significant LOD score was identified on chromosome 7q21, in a region containing >250 genes. Exome sequencing was performed on 2 affected siblings, and variants were called and filtered without taking into account the linkage peak on 7q21. Synonymous, intergenic and intronic variants were removed from further consideration. Variants were filtered based on presence in 1 KG, HapMap, and in the authors’ collection of exome sequences. Heterozygous SNPs were excluded due to the autosomal recessive inheritance mode. This extensive filtering left only 3 homozygous missense variants and only 1 was not present in unaffected family members, a mutation in *GATAD1* that maps to the already identified linkage region on 7q21. *GATAD1* encodes the GATA zinc finger domain-containing protein 1 which is ubiquitously expressed and is thought to bind to a histone modification site that regulates gene expression [79]. Immunohistochemistry with an antibody to GATAD1 revealed an abnormal staining pattern in the proband heart compared to control heart [78]. These data implicate *GATAD1* in the pathogenesis of DCM indicating that exome sequencing can be used to identify novel DCM genes. 

### 3.6. Limitations of WES

While fruitful, exome sequencing does have limitations. There are inherent technical limitations associated with the method. WES requires a capture step, which is limited by design of capture oligonucleotides. Not all genes or exons are adequately annotated and therefore will not be properly included in the methods to capture exons. Furthermore, there can be inconsistencies in capture resulting in poorly covered exons and off-target sequencing. Capture efficiency only approximates 70%–80% in part due to the high GC content of exonic sequence. Probably the most notable limitation is that only 1%–2% of the entire genome is evaluated. Because approximately 85% of described Mendelian mutations occur in the coding regions of genes, it is assumed that Mendelian disease is more likely to be caused by mutations in protein coding exons than in non-coding sequences. However, over a third of Mendelian diseases reported in OMIM have no known molecular basis. It is reasonable to conclude that some of these missing mutations are either structural variants or that they occur in non-coding regions of the genome, exome sequencing is not suited to interrogate either of these possibilities.

Copy number variants (CNVs) are regions, >50 bp in length, that differ from the expected diploid status [80]. CNVs are an important component of genomic variation in humans and some contribute to disease including cardiovascular disease [81,82,83]. A recent study by Norton and colleagues identified a large deletion in *BAG3* in a large multi-generational family with DCM [26]. Whole exome sequencing was performed on 4 of the affected family members and comparative genomic hybridization was performed on the proband to detect copy number variations. The technical limitations of WES prevented identification of the large *BAG3* deletion (>8.7 kb). Algorithms are being created to aid in the use of exome sequencing for the detection of copy number variation [84,85]. However, these methods come with a variety of limitations and cannot detect other types of structural variation such as uniparental disomies or chromosomal rearrangements, both exceedingly important for disease pathogenesis. WGS, as opposed to WES, may offer a better method to detect some structural variants, but may require improved analytic tools for specificity and sensitivity of structural variant detection. 

## 4. WGS as a Tool to Investigate Non-Coding Variation for CVD

Perhaps even more important to understanding disease etiology is the investigation of non-coding variation. Over 98% of the genome is non-coding, and WGS captures nearly 100 fold more of this information compared to WES. However, the interpretation of non-coding variation is currently far more challenging than the interpretation of coding variants. Often referred to as the “dark matter” of the genome, these regions can include microRNAs, long non-coding RNAs, splice variants and regulatory elements that can directly cause or modulate disease phenotypes [37]. miRNAs are important regulators of heart function and recent studies have revealed miRNA misexpression in human cardiac disease and animal models of heart failure [86,87,88]. For example, miR-208 is encoded by an intron within *MYH6*, which encodes α myosin heavy chain and is in close proximity to *MYH7* [87,88]. mir-208 null mice do not hypertrophy in response to cardiac stress and null mice do not upregulate *Myh7* [89,90]. Silencing of miR-208 reduces cardiac remodeling, deterioration of heart function, and improves survival in a rat model of heart failure, while overexpression of miR-208 in cardiomyocytes leads to cardiomyocyte hypertrophy [90,91]. Another miR, miR-1 is the most highly expressed miRNA in the murine heart [88,92]. miR-1 targets HAND2, a transcription factor important for expansion of ventricular cardiomyocytes. Deletion of miR-1 results in 50% perinatal lethality due to ventricular septal defects [88]. The majority of surviving mice exhibit sudden death due to conduction defects. Overexpression of miR-1 in embryonic cardiomyocytes caused thin-walled ventricles leading to death at embryonic day 13.5 [86]. These data underscore the importance of miRNAs in cardiac phenotypes. Variation in these and other non-coding regions of the genome may play a vital role in the disease process. Annotation of the non-coding genome is currently underway to aid in the interpretation of these variants. The ENCODE project (Encyclopedia of DNA Elements) has assigned biochemical functions to 80% of the genome [93]. Only twelve percent of SNPs identified by GWAS as disease-associated are located in the vicinity of a protein-coding region even though SNPs in coding regions are over-represented on SNP arrays. However, over 60% of disease-associated SNPs identified by GWAS lie within functional, non-coding regions, especially in promoters and enhancers [94]. 

### 4.1. WGS Has Greater Sensitivity than WES

WGS is only now emerging as an alternative to WES since higher cost and more complex analysis limited uptake of WGS. Recently, WGS was used to identify a putative causative variant in a family with two children affected by a previously unreported disease defined by cardiomyopathy and progressive muscle weakness [95]. Wang and colleagues performed WES on one sibling and WGS on the other sibling. WES was performed to a depth of 118× with >90% target regions covered by ≥10 reads, while WGS was performed to a depth of 81×. Variants were filtered using ANNOVAR, which relies on frequency and functional variation [96]. After validation with Sanger sequencing and transmission pattern testing, only two genes remained as candidates. One, TAF1L, is homologous to TAF(II)250 and is specifically expressed in testis. The other candidate, *RBCK1*, codes for an E3 ubiquitin-protein ligase. In this family, RBCK1 had two truncating mutations, each inherited from one parent [95]. *RBCK1* was considered a good candidate for both its rarity and the involvement of other ubiquitin-ligase proteins in muscle disease. The WES data set failed to reveal the *RBCK1* variants despite good coverage over the targeted regions including the exons of *RBCK1*. Upon reanalysis, coverage was very low (2 and 4 reads respectively) for the two mutations, with only one read containing a mutation [95]. Further investigation revealed a high GC content surrounding these mutations [97]. Previous studies have also noted that uneven exome coverage has resulted in filtering of disease genes [98]. This study serves as a proof of principle that whole genome sequencing can be used to identify rare Mendelian, cardiomyopathy phenotypes, and, in some instances, may be more sensitive than WES.

### 4.2. Limitations of WGS

**Figure 1:**
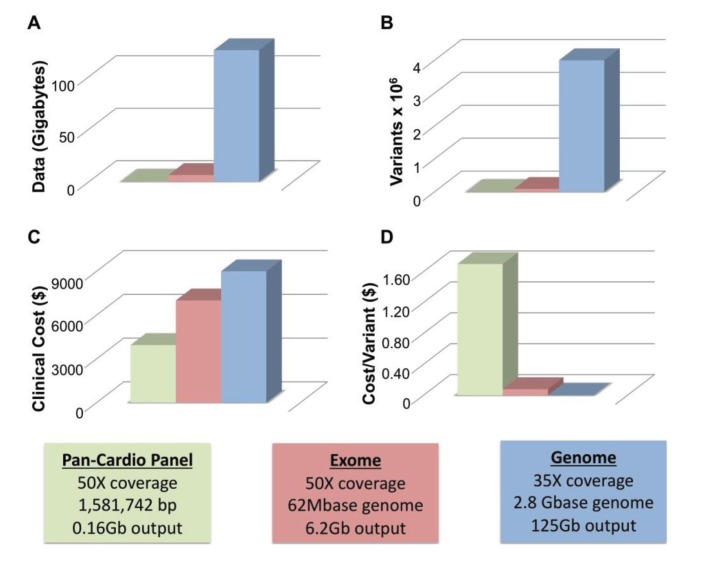
Size and Cost Considerations of Next-Generation Sequencing. (**A**) The amount of data generated by a typical cardiomyopathy gene panel of ~50 genes (green), whole exome sequencing (red) and whole genome Sequencing (blue) is shown; (**B**) The approximate number of variants produced by each method is indicated; (**C**) The Clinical cost of each method ranges from ~$4000 (cardiomyopathy gene panel, green) to ~$9500 (whole genome sequencing, blue); (**D**) The cost per variant is greatly reduced for WGS ($0.002, blue) *versus* WES ($0.08, red) and gene panel-based sequencing ($1.70, green). Boxes indicate parameters used to calculate values in **A**–**D** including coverage, base pairs interrogated and total output.

Two of the major limitations of WGS are size and cost. To achieve average coverage ~35–40× with WGS requires approximately 125 Gb of generated sequencing data. Figure 1A compares the amount of data generated from panel (blue), exome (red) and whole genome sequencing (green). WGS produces an order of magnitude more data than WES. All three technologies call variants proportionate to the amount of data generated with WGS calling ~4 million variants per genome, WES calling ~90 K, and gene panels calling far fewer (Figure 1B). At this time, clinical WGS is more expensive than either WES or panel sequencing at approximately $9000–$9500 for WGS, ~$7000 for WES and ~$4000 for a pan-cardiomyopathy panel. The price for both WES and WGS is considerably more than the cost of a panel. However, this only remains true if a pathogenic mutation is identified in the first panel. The value of each test can be thought of in terms of the cost per variant identified, and with this metric WGS is a better value (Figure 1D). Panel sequencing is ~$1.70, WES is ~$0.08 (8¢) and WGS is ~$0.002 (0.2¢) per variant detected. While only a handful of variants may be germane to identifying the cause of an individual’s primary cardiomyopathy, the other sequence data remains available where it may provide useful guidance for life-style and medical decisions. 

### 4.3. Multi-Pass Filtering Methods Allow for More Efficient Variant Identification

**Figure 2:**
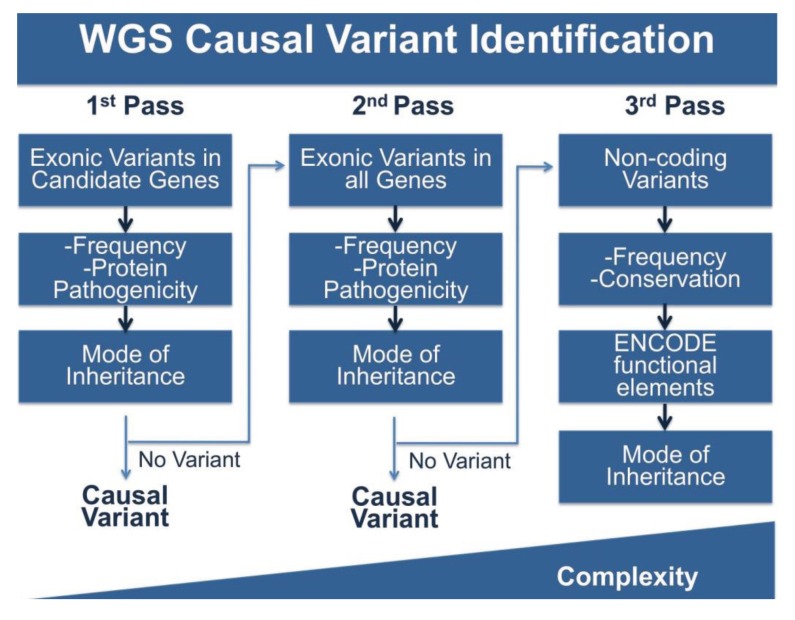
Pipeline for WGS Variant Identification. WGS produces ~4 million variants per genome and requires extensive filtering to identify variants of interest. Shown here is a potential pipeline to identify variants. The first pass of the pipeline entails only reviewing variants in the coding regions of genes of interest and filtering by frequency, protein pathogenicity, and mode of inheritance (segregation in available family members). If no variant is identified, a second pass includes the same filtering steps, but on variants in all coding regions. The third pass includes analysis of non-coding variation using frequency, conservation and ENCODE annotation, along with mode of inheritance. The complexity of analysis increases with each pass.

WGS produces vastly more data than either panels or WES, and this is the double-edged sword of broad based sequence analysis. To cope with this problem we (and others) have adopted stepwise, multi-pass filtering methods (Figure 2). In the first pass, candidate genes are analyzed for the phenotype of interest, in this case cardiomyopathy. Typically, exonic variants in candidate genes are filtered for frequency and for potential protein pathogenicity. A number of tools are freely available that predict the impact of amino acid changes on protein structure and function including PolyPhen-2, GERP, SIFT, PhastCons, Panther and Conseq [72,99,100,101,102,103]. MaxEnt can be used to score the strength of splice site variants [104]. If filtering candidate genes does not produce meaningful variation, the search can be expanded to less attractive candidates or to all rare protein coding variation and finally to non-coding variation. Non-coding variants can be filtered for frequency, however this is more challenging as many frequency databases are biased towards coding regions. Conservation of sequences across multiple species may provide clues about the selection acting on a sequence. The data generated by the ENCODE project will also provide information about variation in functional elements. The complexity of analysis increases with each pass. While this approach approximates panel and WES analysis, it is an improvement in several ways. With this model, WGS only needs to be performed once, while patients that are panel negative will need additional sequencing. In the case of WES, exome capture kits are often updated due to changing gene annotations and the inclusion of newly understood non-coding sequence, requiring retesting with new kits. WGS is less likely to need additional sequencing, and moreover, may provide additional data that over a lifetime can be used to inform not only the primary health concern for which data was collected, but medical choices throughout a patient’s life.

## 5. Incidental Findings and Their Importance for CVD Related Phenotypes

Incidental findings are a concern with all genetic assessment and especially so for WES and WGS. However, incidental findings are not unique to genetic testing and are part of medical decision making for any mode of testing, including imaging and blood tests. WGS may provide many more incidental findings than any other tests available, and this has led to new recommendations for delivering results of incidental findings from genetic research and testing. In 2006, an NHLBI working recommended reporting research results to study participants when the risk of disease is significant and has important health implications including sudden death or considerable risk of morbidity especially when therapeutic interventions are available [105]. In 2013, the American College of Medical Genetics and Genomics (ACMG) made recommendations for reporting incidental findings in exome and genome sequencing [106]. The ACMG recommended that laboratories performing sequencing should identify and report mutations in genes included on their minimal list. Notably, this list includes 24 phenotypes of which a third are cardiovascular disorders for which penetrance is high and clinical interventions are available [106]. Of the 57 genes for which recommendations were made to report incidental findings, more than half (34) were CVD-associated genes.

## 6. Conclusions

In the case of CVD genetic profiling, there are often medical management decisions that can reduce risk. This is the case whether the initial genetic profiling was done for assessing CVD risk or whether the genetic profiling was done to assess risk of other inherited diseases. For example, risks for cardiomyopathy and especially arrhythmias can be managed medically, with increased surveillance or even with device insertion. Risks for developing atherosclerosis or aneurysms can be mitigated through drug or even surgical intervention. Importantly, since CVD disorders can be associated with sudden cardiac death, the capacity to intervene based on genetic risk profiles is evident. With the improvement in genetic databases that are accompanied by robust phenotyping, it should be possible to more accurately predict risk for CVD.
